# Royal Westminster Ophthalmic Hospital

**Published:** 1831-12

**Authors:** 


### ROYAL WESTMINSTER OPHTHALMIC HOSPITAL.

### *Case of Gonorrhœal Ophthalmia.*

John Durant, aged twenty-one, admitted August 10th, 1831; has had gonorrhœa for the last two months. When the discharge from the urethra first appeared, he bought some of Leake's pills, and continued taking two each day for rather more than six weeks: the ardor urinæ and the discharge were then much diminished, but his left eye inflamed. This was a fortnight ago. He still continued taking Leake's pills for three or four days more, but his eye at this time becoming rapidly much worse, he was induced to consult a regular practitioner, who applied four leeches to his left temple, and ordered him to use fomentations. The inflammation increasing, leeches were repeated, at short intervals, to the number of twenty, and one blister has been applied behind his left ear. Two days back, his right eye also became inflamed, and he was ordered, by the same medical gentleman, to apply four leeches to the temple of that side, which he did. This eye, however, getting worse the next day, he became alarmed lest the disease should run the same course as in his left eye, and he therefore came to this hospital.

The eyelids of the left eye are a good deal swollen and inflamed, but, according to his statement, not so much so as they were a few days back; the conjunctiva is exceedingly vascular, chemosed, and granular, and in a great measure overlaps the cornea, which is so extensively injured by the disease, that it will be impossible to restore the sight of that eye. The discharge of matter is still very profuse. During the height of the disease, he suffered from a constant throbbing pain in the eye, but that has subsided with the swelling, and it is perfectly easy now. The disease is only commencing in his right eye: the conjunctiva and sclerotica are a good deal inflamed, but there is no chemosis or swelling of the lids. He complains of severe pricking pain in this eye, which prevented his getting any rest last night. According to the patient's account, the commencement of the disease in his left eye resembled precisely the present state of his right eye; the swelling of the lids and discharge not being much noticed till the fourth or fifth day.

The gonorrhœa is now very slight, having gradually diminished since his left eye inflamed; the glans penis and inner surface of the prepuce are, however, red and excoriated. He is not aware of having done any thing which could have transferred the matter from the urethra to the eye. This is the second gonorrhœa he has had, but the first attack of ophthalmia. His health is much impaired: there is general feverishness, with pain in his joints; he has no appetite, and does not sleep well at night.

Mr. Guthrie applied the nitrate of silver ointment to the right eye, and directed him to use alum lotion to the left. The glans penis is to be kept clean, and constantly smeared with simple dressing. Pulv. Jalap. comp. ℈i. statim.

11th. Says that his right eye is now quite free from the pricking sensation which he complained of yesterday; the redness, however, is not diminished. To foment this eye with tepid water, and continue using the lotion to the left.

12th. So much better that he is surprised to hear that the application of the ointment is again necessary: the inflammation is much less, and he remarks that his sight is stronger. Discharge from the left eye is less profuse. Rept. Ung. Argent. Nitr. oculo dextro.

13th. Continues to improve. The application of the ointment caused him pain for rather more than an hour yesterday; complains of want of rest at night. Pil. Cal. cum Opio, gr. v. nocte. Sulph. Magn. ℥i. mane.

14th. The inflammation of his right eye is now so very trifling that the repetition of the ointment is unnecessary. The disease of the left eye is subsiding; conjunctiva less vascular, and the discharge very much diminished. Gutt. Argent. Nitr. gtt. vi. ad ℥i. oculo dextro.

20th. The drops have been repeated regularly every morning to his right eye, but have not removed the slight inflammation of the conjunctiva of the lids. There is little or no discharge from the left eye now; the chemosis has quite subsided, and the vascularity of the conjunctiva has very much diminished: cornea is healing better than could be expected, but is disorganized and opaque; he says, however, that he can see his hand when it is passed close before this eye. The discharge from the urethra has quite ceased, and the glans penis is well. A drop of Vinum Opii to be applied to each eye.

September 2d. The right eye is now perfectly well, and the left is much improved in appearance, and causes no inconvenience. To continue the drops to the left eye.

It may, perhaps, appear unnecessary to some of our readers to add to the evidence we have already adduced in favor of the plan of treatment adopted by Mr. Guthrie in cases of gonorrhœal ophthalmia: there are still, however, some practitioners who doubt the safety of this treatment, and deny its efficacy. An accumulation of proof may tend to remove their scepticism. Once more we must point out the necessity of applying the nitrate of silver ointment according to Mr. Guthrie's directions.* We have known surgeons frequently fail in relieving the symptoms, in consequence of the ointment being too weak, or from the ineffective mode in which it was applied, and who, after a strict adherence to Mr. Guthrie's plan, have acknowledged the value, and almost certain efficacy, of the remedy, in the common purulent as well as gonorrhœal ophthalmia.

* London Med. and Phys. Journal, September 1828.

